# 1517. Sexual Network Characteristics of MSM with Detectable Viremia Living with HIV in Lima, Peru

**DOI:** 10.1093/ofid/ofad500.1352

**Published:** 2023-11-27

**Authors:** Carlyn L Harris, Eddy Segura, Jessica Gutierrez, Jordan Lake, Cherie Blair, Jesse Clark

**Affiliations:** Emory University School of Medicine, Atlanta, Georgia; UCLA, Los Angeles, California; Via Libre, Lima, Lima, Peru; University of Texas Health Science Center at Houston, Houston, TX; David Geffen School of Medicine at UCLA, Los Angeles, California

## Abstract

**Background:**

In Lima, Peru, the HIV epidemic is concentrated among men who have sex with men (MSM) and transgender women (TW). Few data exist on the sexual network characteristics of MSM with detectable HIV-1 viremia, yet these networks have important implications to control transmission. We report data on frequency of sero-discordant, condomless anal intercourse (CAI) among MSM in Lima with detectable viremia.

**Methods:**

This cross-sectional secondary analysis includes MSM screened for a STI trial in Lima, Peru between June 2022 and January 2023. Participants completed questionnaires on demographics, sexual identity and role, and sexual network characteristics. Participants were screened for HIV infection and those who tested positive were included and HIV-1 RNA was measured. Descriptive and comparative statistics are reported for those with detectable and undetectable viremia.

**Results:**

665 MSM with HIV were included. 153 (23%) had detectable ( >200 copies/mL) viremia. Among those with detectable viremia, the median (IQR) age was 29 (25, 35) years. 70% identified as homosexual and 57% identified their sexual role during intercourse as “*moderno*” or “*versatil*”. The median (IQR) number of sexual partners in the last 3 months was 15 (5,20). 55/153 (35.6%) of the detectable group were aware of their HIV diagnosis, 33/55 (60%) reported being on anti-retroviral therapy (ART) and 15/32 (47%) believed they were virally suppressed. Among all participants living with HIV, 499/662 (75%) were aware of their HIV status, 469/499 (94%) reported to be on ART and 436/469 (93%) were virally suppressed. 147/153 (96%) of men with detectable viremia reported sero-discordant CAI with at least one of their last three sexual partners, and 106/144 (74%) reported this with all three of their last partners. In the undetectable group, 302/489 (62%) reported sero-discordant CAI with all three of their last partners (p < 0.01).

**Conclusion:**

In this sample of MSM living with HIV in Lima, Peru, almost all men with detectable HIV-1 viremia reported sero-discordant CAI, more often than those with undetectable viremia. The primary gap in the HIV care cascade was participant awareness of their HIV status, suggesting that broader HIV testing and improved access to resources for secondary HIV prevention are key objectives in Peru.

**Disclosures:**

**Jordan Lake, MD**, Gilead Sciences: Grant/Research Support|Theratechnologies: Advisor/Consultant

